# Caught in the Net: Perineuronal Nets and Addiction

**DOI:** 10.1155/2016/7538208

**Published:** 2016-01-19

**Authors:** Megan Slaker, Jordan M. Blacktop, Barbara A. Sorg

**Affiliations:** Department of Integrative Physiology and Neuroscience, Washington State University, Vancouver, WA 98686, USA

## Abstract

Exposure to drugs of abuse induces plasticity in the brain and creates persistent drug-related memories. These changes in plasticity and persistent drug memories are believed to produce aberrant motivation and reinforcement contributing to addiction. Most studies have explored the effect drugs of abuse have on pre- and postsynaptic cells and astrocytes; however, more recently, attention has shifted to explore the effect these drugs have on the extracellular matrix (ECM). Within the ECM are unique structures arranged in a net-like manner, surrounding a subset of neurons called perineuronal nets (PNNs). This review focuses on drug-induced changes in PNNs, the molecules that regulate PNNs, and the expression of PNNs within brain circuitry mediating motivation, reward, and reinforcement as it pertains to addiction.

## 1. Introduction

Repeated exposure to drugs of abuse creates persistent drug-related memories that may be the foundation of the chronic relapse problem. Memories for the drug, drug-related cues, and drug-related contexts are strengthened over time with repeated drug use. Disruption of these drug-related memories by targeting key neuroplastic events required for memory stabilization may lead to suppression of relapse. Recently, attention has shifted from targets located on the pre- and postsynaptic cells and astrocytes to targets located in the extracellular matrix (ECM) within brain regions important for drug-taking behavior and drug-related memories. This shift has contributed to the advancement from a tripartite synapse theory ((1) presynapse; (2) postsynapse; (3) astrocyte) to a tetrapartite synapse theory, which includes the ECM [1, 2].

Perineuronal nets (PNNs) are net-like structures composed of aggregations of ECM molecules. PNNs surround a subpopulation of neurons, and, in many brain regions, they surround primarily GABAergic, parvalbumin- (PV-) containing, fast-spiking interneurons [3–5]. They are most commonly identified by staining with the plant lectin,* Wisteria floribunda* agglutinin (WFA), although other methods have also been used [3, 6, 7]. PNNs are rich in chondroitin sulfate proteoglycans (CSPGs; primarily of the lectican family, including aggrecan, brevican, and versican) and hyaluronan (HA [8, 9]). Other molecules in the PNN are tenascins and link proteins (HAPLNs [10, 11]). Many plasticity-regulating molecules bind to or interact with the PNN, including semaphorin-3A, brain-derived neurotrophic factor (BDNF), and Otx2 [12–14]. PNNs have been heavily studied for their contributions to critical period plasticity within the visual system, motor system, and somatosensory system [15–17]. Despite the emerging significance of the role of PNNs in a variety of types of plasticity, very little is known about the plasticity of PNNs and PNN-surrounded neurons induced by drugs of abuse. Much less is known about how PNNs regulate function of their underlying neurons within brain regions that contribute to aberrant motivation, reward, and reinforcement underlying addiction. Understanding the contribution of PNNs to plasticity, and eventually harnessing this plasticity, may lead to therapeutic intervention for many pathologies, including addiction. Here we discuss how PNNs may regulate plasticity of brain regions heavily implicated in memory, reward, and addiction.

## 2. PNN and Memory

The brain regions that are important for acquiring and maintaining a fear-conditioned memory overlap with brain regions that are important for acquiring and maintaining a drug-related memory [18]. Both types of memory consist of context- and cue-related information and motivated behavior. The most common method to examine a fear-conditioned memory is to train an animal to associate the presentation of a cue, typically a tone, with a footshock. This memory can be extinguished by repeated presentation of the cue without presence of the shock. Interestingly, in rats younger than 3 weeks, extinction training of the fear-conditioned memory is capable of rewriting or potentially erasing the original memory trace, whereas, in adult animals, extinction training creates a new memory but does not erase the original memory [19]. PNNs are important for the acquisition and maintenance of a fear-conditioned memory. Juvenile animals do not have PNNs but adult animals do. Thus, the developmental timetable of PNNs within the basolateral amygdala (BLA), a region of the brain critical for fear conditioning, mirrors the loss in ability of extinction training to override the original memory trace [19]. Not only does the appearance of PNNs coincide with this behavioral change, but removing PNNs from the BLA by local administration of chondroitinase-ABC (Ch-ABC), a bacterial enzyme used to degrade components of the ECM and PNN, reverts the behavior of an adult animal back to the behavior of a juvenile animal [19]. These findings suggest that PNNs contribute to the maintenance of strong fear memories.

PNNs within the hippocampus and medial prefrontal cortex (mPFC) also play a role in fear conditioning [20]. Removal of PNNs within the hippocampus impairs context-induced reinstatement of fear conditioning, whereas removal of PNNs within the mPFC impairs cue-induced reinstatement of fear conditioning [20]. Collectively, these findings suggest that PNNs contribute to the development and maintenance of a fear-conditioned memory; however, the role they play in specific types of memory maintenance (e.g., cue- or context-dependent memory) appears to be brain region-dependent. Each of the studies used a global disruption method to target not only the PNN, but all of the ECM. Future studies will be needed to more specifically target the PNN and verify the effects of these studies.

## 3. PNNs and Reward-Related Circuitry

Examination of brain regions with relatively high PNN expression may provide insight into the neuroanatomical function of PNNs, including brain regions implicated in the circuitry of motivation, reward, and reinforcement. Brain regions that have been heavily focused on include the amygdala, hippocampus, and regions of the PFC because they are critical for activation of goal-directed behavior, memory, and addiction [21–23]. Significant PNN expression has been reported in over 100 brain regions of the rat, ranging from the rostral cortex to the spinal cord, but few studies have characterized the expression of PNNs in brain regions implicated in addiction: the prefrontal cortex (PFC), amygdala, hippocampus, cerebellum, striatum, ventral pallidum, and hypothalamus [3, 24, 25].

### 3.1. Prefrontal Cortex

The PFC contains numerous distinct regions that contribute to addiction. In general, the dorsomedial PFC (dmPFC) facilitates drug seeking, while the ventromedial PFC (vmPFC) impedes drug seeking [18, 26–29]. Within both of these regions, PNNs are primarily colocalized with PV-containing, GABAergic interneurons, which are well conserved across species including rat, primate, and human [3, 6, 30–36]. This population of PV-containing interneurons regulates pyramidal neuron output from the PFC and regulates gamma oscillations important for working memory and optimal cognitive processing [37–41]. In addition to the mPFC, the orbitofrontal (OF) region of the PFC has consistently been implicated in the representation of value of a reinforcer and in value-based decision-making [22, 42]. Aberrant OF activity has been observed following drug exposure and is thought to mediate compulsive drive and craving in humans and cue-induced reinstatement in rodents [43, 44]. The OF exhibits relatively robust PNN expression and many PNN components within the OF increase following intermittent binge ethanol exposure [24, 45]. Collectively, PNNs across subregions of the PFC are affected by drug exposure and may provide a target to specifically modify circuitry of motivated behavior.

### 3.2. Amygdala

The amygdala is critical for emotional processing, reward valuation, and learning [22, 46]. The amygdala is well situated between the PFC and the ventral striatum to provide key neurocircuitry mediating both stress- and cue-induced reinstatement of drug-seeking behavior [22, 47]. Studies on PNN expression differ between species within the amygdala. Early studies examining the amygdala of the* rodent* reported relatively low PNN expression [24, 25]; however, a more recent study examining the BLA of* humans* reported significant PNN expression [48]. Despite these conflicting reports, PNNs within the amygdala of the rodent have been directly implicated in both fear and addiction behavior (see PNNs in memory and addiction sections).

### 3.3. Hippocampus

The hippocampus is importantly involved in memory and has also been implicated in reward circuitry regulating drug-seeking and drug-taking behavior [49–55]. The hippocampus expresses significant levels of PNNs [24, 25]. Both PV and PNNs colocalize within basket cells in the CA1 and CA3 regions and the granule cell layer of the dentate gyrus [56–58]. This colocalization does not appear within the CA2 region, where PNNs surround cells that are presumed to be non-GABAergic pyramidal neurons [59]. The expression of PNNs within the hippocampus provides an interesting region to study the differences among the subpopulations of PNN-surrounded neurons (PV+, PV−, and pyramidal) and how each of these subpopulations contributes to memory and drug-seeking/taking behavior.

### 3.4. Cerebellum

Although not traditionally viewed as part of addiction-related circuitry, the cerebellum is activated during episodes of drug craving and exhibits drug-induced plasticity [60–63]. In the cerebellum, PNNs are found primarily around large, excitatory neurons of the deep cerebellar nuclei, while granule cell layer Golgi neurons of the cerebellar cortex express PNNs in comparatively low numbers and do not colocalize with PV [24, 25, 64, 65]. Exposure to cocaine increases the proportion of PNNs expressing strong WFA staining intensity in the deep cerebellar medial nucleus, which is a PNN-rich region characterized by large projection neurons [63].

### 3.5. Other Regions

The striatum is heavily implicated in reward and motivated behaviors and consists of the nucleus accumbens, caudate nucleus, and putamen. Low levels of sporadic PNN staining have been reported in all three regions of the striatum in the rat [24, 25]; in contrast, in the mouse, significant PNN expression has been reported throughout the striatum [66].

The ventral pallidum is essential for the integrative component of the limbic system contributing to motivated behavior and drug seeking [47, 67]. The ventral pallidum exhibits robust PNN expression [24], making it a promising, yet greatly understudied, brain region with regard to the role of PNNs in motivated behavior.

Finally, the lateral and anterior hypothalamus are important for motivated behaviors and contain strong PNN expression [24, 68–71]. However, PNNs in the lateral hypothalamus are PV-containing glutamatergic neurons rather than GABAergic neurons, while PNNs in the anterior hypothalamus are calretinin/enkephalin-containing neurons rather than PV-containing neurons [70, 71].

Understanding the functional plasticity of PNNs throughout the circuitry underlying motivated behaviors is in its infancy but shows promise in delineating drug-induced changes in circuit plasticity. Based on regional differences in the expression of PNNs, additional work is needed to address whether drugs of abuse alter expression of PNNs in these different regions and whether modifying PNNs after drug exposure or other behavioral manipulations could mitigate drug-induced plasticity. Next, we examine the current research addressing these questions.

## 4. PNNs and Addiction

While the expression of PNNs varies across circuitry mediating reward, few studies have investigated the role of PNNs in drug- and reward-related behaviors. In 2010, Van Den Oever and colleagues [27] trained rats on a heroin self-administration task and measured levels of PNN components within the mPFC following cue-induced reinstatement. Protein and mRNA levels of brevican, tenascin-R, and HA were decreased following a period of* forced abstinence* or* extinction training* from animals that self-administered heroin compared to levels from animals that self-administered saline. However, following cue-induced reinstatement of heroin self-administration, brevican returned to control levels. These findings suggest that PNN components dynamically respond to environmental cues, possibly via a reduction in ongoing MMP activity or a redistribution of PNN components [27]. Following cue-induced reinstatement, the frequency of spontaneous inhibitory postsynaptic currents (sIPSC) was increased compared to animals not exposed to the cues; the amplitude and decay-time constant of sIPSC did not change. This finding suggests that the functioning of GABAergic interneurons within the mPFC is increased in the presence of drug-associated cues and may be related to the changes that are occurring within PNNs that envelop these interneurons. Future studies are needed to explore these possibilities.

In the second study examining the role of PNNs in drug-seeking behavior, Xue et al. [72] removed PNNs using Ch-ABC within the BLA or central amygdala prior to extinction training of morphine-induced or cocaine-induced conditioned place preference (CPP). They found that PNN removal enhanced extinction training, resulting in decreased reinstatement of CPP behavior. Additionally, removal of PNNs within the BLA or central amygdala prior to extinction training of heroin self-administration also enhanced extinction training and decreased spontaneous recovery. In addition to behavior, markers of plasticity were examined, including BDNF and GluR1–3. Interestingly, removal of PNNs without extinction training or extinction training alone resulted in a similar increase in protein levels of BDNF, GluR1, and GluR2, whereas removal of PNNs* plus* extinction training resulted in an even greater increase in BDNF, GluR1, and GluR2 levels compared with no extinction training in animals with intact PNNs [72]. This study suggests that PNN removal may augment metaplastic conditions necessary to enhance extinction training of drug-induced CPP. This finding is consistent with previous studies demonstrating that removal of PNNs promotes experience-dependent plasticity [12, 15, 17, 73]. Future studies are needed to explore more specific methods of targeted PNN disruption and exploration of changes that are occurring in the PNN components following drug exposure.

Finally, recent work from our laboratory has examined the role of PNNs within the mPFC on the acquisition and maintenance of a cocaine-induced CPP memory. Removal of PNNs using Ch-ABC within the dmPFC impaired acquisition and reconsolidation of a CPP memory [36]. Interestingly, this effect was specific for the dmPFC because removal of PNNs from the vmPFC had no effect on acquisition of the task. Additionally, removal of PNNs within the dmPFC prior to extinction training had no effect on extinction or subsequent cocaine-induced reinstatement of CPP. In parallel with the observed behavioral decrease following PNN removal, we also found a decrease in the number of PNN-surrounded neurons that were positive for the immediately early gene c-Fos, but only in animals that displayed impaired memory. These results suggest that the acquisition and reconsolidation processes are impaired following PNN removal from the dmPFC, which may be a result of impaired activation of PNN-surrounded neurons. Additionally, we found that removal of PNNs results in hyperexcitability in pyramidal cells within the dmPFC. Removal of PNNs using Ch-ABC decreased the frequency of mIPSCs on pyramidal neurons and the number of action potentials. Interestingly, cocaine-induced CPP decreased both the frequency and amplitude of mIPSCs on pyramidal neurons. These findings suggest that ECM disruption and PNN removal may occlude cocaine-induced adaptations in the dmPFC.

## 5. Regulation of PNNs by Molecules Involved in Addiction

Under normal physiological circumstances, the ECM and PNNs have many endogenous regulators that can remodel PNNs to potentially allow for drug-induced plasticity to occur. Many molecules regulate components of the PNN and may therefore contribute to drug-induced plasticity. The two molecules we will focus on here are matrix metalloproteinases (MMPs) and BDNF.

### 5.1. Matrix Metalloproteinases

MMPs are a family of proteolytic enzymes (25+ members) that degrade components of the ECM, including those found within PNNs. MMPs are synthesized in an inactive proform, released from the cell following neuronal stimulation, and cleaved into an active MMP form. Many MMPs can act on PNN components and have been implicated in drug-related plasticity.

Two and 24 hours following the fifth consecutive day of noncontingent methamphetamine exposure, protein and activity levels of MMP-2 and MMP-9 increased within the nucleus accumbens and PFC of rats [74]. Additionally, following reinstatement of cocaine-induced CPP, activity levels of MMP-9 within the mPFC peaked 1–3 hours following reinstatement and returned to normal levels by 24 hours; however, MMP-2, MMP-3, and MMP-9 levels within the dorsal hippocampus and MMP-2 and MMP-3 levels within the mPFC were unchanged [75]. Similarly, a 15 min cue-induced reinstatement of cocaine or nicotine self-administration, a 45 min cocaine-induced reinstatement, or a 2-hour extinction session following cocaine or nicotine self-administration increased activity of MMP-2 and MMP-9 within the nucleus accumbens core [76]. Additionally, a 15 min cue-induced reinstatement of heroin self-administration increased MMP-2 and MMP-9 activity in the nucleus accumbens core [76]. In contrast to stimulant exposure, repeated alcohol exposure decreased MMP-9 activity within the hippocampus and PFC of rats after 2, 4, or 6 days [77]. These studies suggest that drugs of abuse change the activation of MMPs, which may allow for remodeling of the ECM and PNNs. These studies also suggest that the direction of changes is dependent on the type of drug, stimulant or depressant, although it remains unknown whether the duration and dose of drug exposure might also contribute to the opposite outcomes in MMP activation state.

To test the hypothesis that MMP activation is contributing to drug-seeking behavior, the effect of impairing MMPs on this behavior has been investigated. Impairment of MMP-2 and MMP-9 in knockout mice or administration of a nonspecific MMP inhibitor into the PFC reduced sensitization and disrupted methamphetamine-induced CPP [74]. Additionally, administering a nonspecific MMP inhibitor, FN-439, into the lateral ventricles blocked cocaine-induced reinstatement of CPP, reduced alcohol intake following acute withdrawal, and attenuated cue-induced heroin seeking [27, 78, 79]. These studies provide evidence that MMPs are involved in the neural response to drugs of abuse; however, additional studies need to investigate the effect of overexpression of MMPs on drug-seeking behavior or endogenous inhibitors of MMPs, such as tissue inhibitors of MMPs (TIMPs [74]). In addition, other ECM remodeling enzymes need to be investigated, including members of a Disintegrin and Metalloproteinase with Thrombospondin Motifs (ADAMTs) family of enzymes [80].

### 5.2. Brain-Derived Neurotrophic Factor

BDNF is important for many types of plasticity, including drug-induced plasticity. For example, global upregulation of BDNF enhanced extinction training that in turn decreased drug-seeking behavior [81]. Since BDNF has been implicated in a number of types of plasticity, it is perhaps not surprising that it also affects the expression of PV and PNNs, but the relationship between BDNF and PNN expression is far from clear. Overexpressing BDNF* in vivo* accelerates the appearance of PV+ synapses during development, which is used as a marker for neuronal maturation [82]. As demonstrated by Xue et al. [72], the removal of PNNs increased BDNF levels in the BLA in animals exposed to morphine, which suggests that PNN removal modifies how BDNF responds to this drug of abuse. Future research is required to determine detailed interactions between BDNF and its impact on PNNs. Interestingly, MMP-9 is thought to promote the conversion of pro-BDNF to the biologically active BDNF [83], and BDNF also induces MMP-9 expression* in vitro* [84].

## 6. Caveats and Future Directions

The role of PNNs in the creation and maintenance of drug-related memories and drug-seeking behavior is in its infancy. A foundation has been formed, but future studies will need to focus on how PNNs are modulated by different drugs of abuse (contingent and noncontingent) within brain regions important for motivation, reward, and reinforcement.

One important issue to consider is the methodology used to study the role of PNNs. Most studies, including our own, have used the bacterial enzyme, Ch-ABC, to degrade CSPG chains found both within the loose ECM and within the PNNs. This manipulation confounds results due to lack of specificity for the PNN. Knockout mice have been created that lack Hapln-1 within the central nervous system. Halpn-1 knockout mice die at birth because a total lack of Halpn-1 leads to cardiac defects [12]. To overcome this issue, Halpn-1 can be expressed under the control of type II collagen cartilage-specific promoter and enhancer, which allows Halpn-1 to be expressed in cartilage but not in the central nervous system [12]. These conditional knockout mice have attenuated PNNs and display similar ocular dominance plasticity as wild-type juvenile mice prior to the appearance or development of PNNs [12]. Additionally, this plasticity is similar to adult animals treated with Ch-ABC [12]. These results suggest that Halpn-1 within the central nervous system is necessary for proper formation of PNNs and for limiting plasticity following the critical period in development. Currently, this knockout exists only in mice. siRNA, shRNA, or morpholinos are other strategies to target Halpn-1 in other species. A second methodology consideration is how PNNs are visualized. Most PNNs are labeled using WFA, although other plant lectins also bind to and label sugar groups present in PNNs [6]. Using an antibody for a specific component of PNNs, for example, aggrecan, is one method to visualize the aggregation of the CSPGs within the PNN.

A second consideration is the length of time between PNN removal and examination of behavioral or cellular changes. For example, removal of PNNs with Ch-ABC decreases WFA staining of PNNs for a period of days, but the reappearance of PNNs is gradual and is expected to create various stages of plasticity in the intervening period until PNNs are fully restored [36]. Thus, the direction and extent of behavioral and cellular changes are likely to be different depending upon the extent of removal and when they are examined following removal.

A third important consideration is how well conserved PNN expression is across species, from rodents to humans. Subpopulations of PNN-surrounded neurons differ across species and brain regions. In some studies, PNN expression around cortical pyramidal neurons is found in humans and primates but not in rodents [85, 86]. However, in other studies, PNN expression around glutamatergic pyramidal neurons has been reported in rodents [87]. PNN expression in the amygdala has also been found primarily around* astrocytes* in the human, but primarily around* neurons* in the monkey and rat [48]. Taken together, these results suggest that PNN expression within the brain is heterogeneous in both a species- and brain region-specific manner. Further comparative anatomical characterization of PNN expression across mouse, rat, monkey, and human will benefit the translational impact of this research.

## 7. Conclusions

While the role of PNNs in motivation, reward, and reinforcement is only beginning to be understood, recent findings indicate that exposure to drugs of abuse alters PNNs and that these structures are necessary for creating and/or maintaining drug-related memories. Future studies will be needed to provide a detailed understanding of the dynamic cellular changes occurring in PNNs and PNN-surrounded neurons to determine how these structures and neurons contribute to maintaining drug-related memories. In summary, PNNs and PNN-surrounded neurons may serve as novel targets for diminishing memories that drive relapse to drugs of abuse.
